# Improved accuracy of myocardial blood flow quantification by first pass perfusion MR when corrected for steady state T1 relaxation

**DOI:** 10.1186/1532-429X-15-S1-P85

**Published:** 2013-01-30

**Authors:** Niloufar Zarinabad, Eva Sammut, Tobias Voigt, Gilion Hautvast, Marcel Breeuwer, Reza Razavi, Eike Nagel, Valentina O Puntmann, Amedeo Chiribiri

**Affiliations:** 1 Imaging Sciences and Biomedical Engineering, Kings College London, London, UK; 2 Imaging Systems, MR, Philips Healthcare, Best, the Netherlands; 3 Biomedical Engineering,Biomedical Image Analysis, Eindhoven University of Technology, Eindhoven, the Netherlands; 4 Clinical Research Europe, Philips Research, Aachen, Germany; 5 Healthcare Incubators, Philips Innovation Group, Eindhoven, the Netherlands

## Background

**Figure 1:**
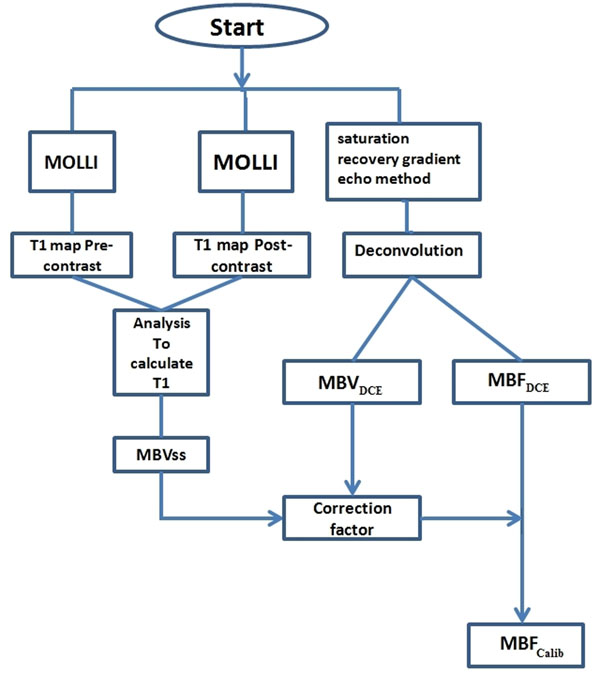
Schematic of the calibration method. This method combines the bookend technique with an automatic quantification algorithm to improve the accuracy of MBF estimates.

Quantification of myocardial blood flow (MBF) on first-pass dynamic contrast enhanced (DCE ) MR perfusion imaging has been performed by several different methods. However MBF estimates are dependent on the analysis model used, as the absolute concentration of the contrast agent during first pass is not known. To improve the accuracy of the MBF estimates calibration is required. We propose a novel approach for the quantification of MBF based on patient-specific calibration using steady state T1 relaxation values (1) (figure 1).

## Methods

T1 weighted images were acquired before and after contrast injection to perform steady state measurements of myocardial blood volume (MBVss) (2): MBVSS = (1/T1post contrast -1/T1pre contrast) tissue / (1/T1post contrast -1/T1pre contrast) blood.

Based on the first pass perfusion data, MBVDCE and MBFDCE were obtained using Fermi deconvolution. The calibration factor (CF) was calculated as CF= MBVss / MBVDCE and used to calibrate MBF values (MBFcalib) according to: MBFcalib = MBFDCE *CF.

Perfusion data were obtained from five patients using a dual-bolus injection of 0.075mmol/kg Gadobutrol (Gadovist, Bayer, Germany) injected at 4ml/minute followed by a 20 ml saline flush using a Philips Achieva 3T (TX) system, equipped with a 32-channel cardiac phased array receiver coil (Philips, Best, Netherlands). Hyperaemia was induced with adenosine administered at 140 mcg/kg/min. First-pass perfusion images were acquired using a saturation recovery gradient echo method (TR/TE 3.0ms/1.0ms, flip-angle 15°; effective k-t SENSE acceleration 3.8, spatial resolution 1.2x1.2x10mm, saturation-recovery delay 120 ms). T1 images were acquired using a cardiac triggered Modified Look Locker Inversion Recovery (MOLLI) sequence(slice thickness 8 mm, FOV 388x320mm2, matrix 216x216, 11 different TI, range 90ms-3s). T1 was estimated through fitting of a three parameter exponential model SI(t) = A - B exp( t/T1*) and Look-Locker correction T1 = T1*((B/A) - 1).

## Results

**Figure 2:**
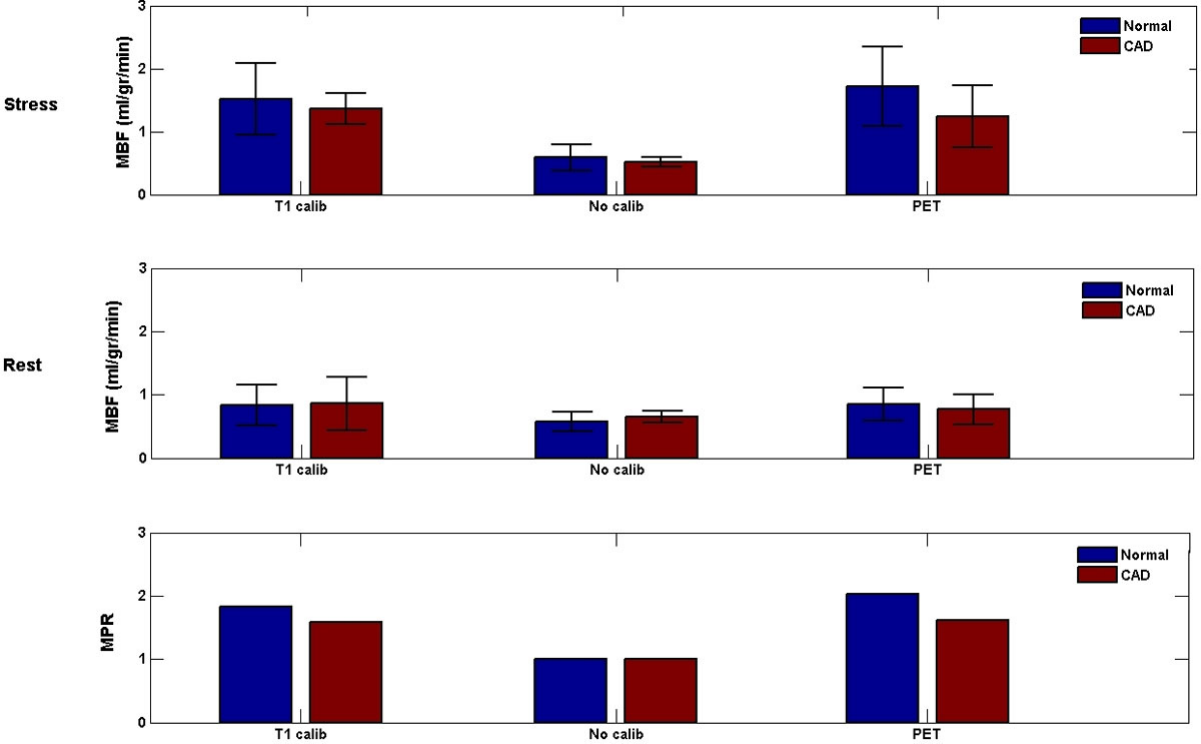
Comparison between MBFcalib (ml/ gr/min) values determined by the novel T1 calibration algorithm (T1 calib), the MBF values obtained from standard non calibrated algorithm (no calib) and reference values from literature in normal (Blue) and ischemic (red) myocardial regions. The calibrated measurements agree with literature values.

The bar plot in figure 2 compares the rest and stress mean MBFcalib values with the mean MBFDCE values and the reference perfusion values reported in the literature (3) in normal and ischaemic myocardial regions. The mean stress values of MBFcalib for both normal and abnormal regions (1.35±0.24 ml/gr/min for ischemic and 1.51±0.56 ml/gr/min for normal region) are in closer agreement with stress PET values (1.24±0.49 ml/gr/min for ischemic and 1.73±0.63) compared with the MBFDCE values obtained from non calibrated data (0.58±0.207 ml/gr/min for ischemic and 0.65±0.070for normal).

## Conclusions

The measurements of quantitative MBF agree closely with previously published gold-standard measurements by PET after calibration is performed. The addition of T1 steady state measurements to DCE measurements of MBF improve the accuracy of the measurement as inter-subject variability is taken into account.

## Funding

The authors acknowledge financial support from the Department of Health via the National Institute for Health Research (NIHR) comprehensive Biomedical Research Centre award to Guys and St Thomas NHS Foundation Trust in partnership with Kings College London.
